# Unilateral hindlimb ischaemia‐induced systemic inflammation is associated with non‐ischaemic skeletal muscle inflammation

**DOI:** 10.1113/EP091901

**Published:** 2024-06-18

**Authors:** William S. Evans, Gabriel S. Pena, Beata Gelman, Sarah Kuzmiak‐Glancy, Steven J. Prior

**Affiliations:** ^1^ Department of Kinesiology University of Maryland School of Public Health College Park Maryland USA; ^2^ Baltimore Veterans Affairs Geriatric Research Education and Clinical Center and Research and Development Service Baltimore Maryland USA

**Keywords:** angiogenesis, macrophage, muscle atrophy, neutrophil

## Abstract

**Abstract:**

Skeletal muscle atrophy and dysfunction commonly accompany cardiovascular diseases such as peripheral arterial disease and may be partially attributable to systemic inflammation. We sought to determine whether acute systemic inflammation in a model of hindlimb ischaemia (HLI) could affect skeletal muscle macrophage infiltration, fibre size, or capillarization, independent of the ischaemia. Eight‐week‐old C57BL/6 male mice underwent either Sham or HLI surgery, and were killed 1, 3, or 7 days post‐surgery. Circulating inflammatory cytokine concentrations were measured, as well as immune cell infiltration and morphology of skeletal muscle from both limbs of HLI and Sham mice. In HLI compared with Sham mice at day 1, plasma interleukin‐1β levels were 216% higher (0.48 ± 0.10 vs. 0.15 ± 0.01 pg/μL, *P* = 0.005) and decreased by day 3. This was followed by increased macrophage presence in muscle from both ischaemic and non‐ischaemic limbs of HLI mice by day 7 (7.3‐ and 2.3‐fold greater than Sham, respectively, *P* < 0.0001). In HLI mice, muscle from the ischaemic limb had 21% lower fibre cross‐sectional area than the non‐ischaemic limb (724 ± 28 vs. 916 ± 46 μm^2^, *P* = 0.01), but the non‐ischaemic limb of HLI mice was no different from Sham. This shows that HLI induces acute systemic inflammation accompanied by immune infiltration in both ischaemic and remote skeletal muscle; however, this did not induce skeletal muscle atrophy in remote muscle within the 7‐day time course of this study. This effect of local skeletal muscle ischaemia on the inflammatory status of remote skeletal muscle may signal a priming of muscle for subsequent atrophy over a longer time course.

**Highlights:**

- **What is the central question of this study?**
  Does hindlimb ischaemia‐induced inflammation cause acute immune, inflammatory and morphological alterations in remote non‐ischaemic skeletal muscle?
- **What is the main finding and its importance?**
  Hindlimb ischaemia induced systemic inflammation with subsequent neutrophil and macrophage infiltration in both ischaemic and non‐ischaemic skeletal muscle; however, morphological changes did not occur in non‐ischaemic muscle within 7 days. These immune alterations may have functional implications that take longer than 7 days to manifest, and subsequent or prolonged systemic inflammation and immune infiltration of muscle could lead to morphological changes and functional decline.

## 1 INTRODUCTION

Cardiovascular diseases (CVDs) such as heart failure and peripheral arterial disease frequently result in reduced functional capacity, low aerobic capacity and exercise intolerance (Jurgens et al., 2022). These alterations are due, in part, to changes in skeletal muscle rather than a cardiovascular defect per se (Kitzman et al., 2017). In several CVDs, skeletal muscle is shown to have fibrosis, fibre atrophy, glycolytic fibre type shifts, lower capillarization, and reduced mitochondrial function (Adams et al., 2017; Duscha et al., 1999; Kitzman et al., 2016, 2017; Poole et al., 2018; Vescovo et al., 1998); however, the underlying causes of these skeletal muscle changes are not well understood.

Systemic inflammation is hypothesized to contribute to skeletal muscle dysfunction in CVDs (Middlekauff, 2010; Seiler et al., 2016). One possible explanation is that inflammation disrupts skeletal muscle angiogenesis and directly impacts muscle fibres through altered recruitment of skeletal muscle macrophages. Some studies show that following tissue ischaemia, macrophages contribute to tissue healing and angiogenesis (Mirza et al., 2009; Ochoa et al., 2007), while other studies show that the elevated or persistent presence of inflammatory macrophages can actually reduce myoblast proliferation, increase tissue fibrosis and induce premature myoblast differentiation (Chen et al., 2007; Miller et al., 1988). Specifically, pro‐inflammatory macrophages are typically responsible for the early response and propagating the inflammatory stimulus, after which there is a transition to anti‐inflammatory macrophages to resolve inflammation and contribute to tissue repair (Mosser & Edwards, 2008). Thus, timely infiltration of skeletal muscle macrophages following ligation of the femoral artery to induce hindlimb ischaemia (HLI) may be crucial to restore blood flow to the ischaemic limb via angiogenesis and arteriogenesis. Though the HLI model has been primarily used to study the effects of local ischaemia on local tissue repair, and murine hindlimb ischaemia is an oft‐used model of peripheral arterial disease used to study the direct effects of ischaemia, a previous study showed that HLI also resulted in remote changes to carotid artery repair (Sorrentino et al., 2018) suggesting there are functional ramifications of systemic inflammation on remote tissues. However, studies have not followed up on this finding, and to our knowledge the HLI model has not been used to study the similarities and differences between the local effects of ischaemia and remote effects of systemic inflammation resulting from the local ischaemia, specifically on skeletal muscle macrophages, angiogenesis and skeletal muscle itself.

Therefore, the purpose of this study was to determine the 7‐day time course of the effects of an acute inflammatory stimulus on skeletal muscle from both the ischaemic and non‐ischaemic limbs of mice subjected to HLI. Using this model, skeletal muscle of the ischaemic limb is subjected to both inflammation and ischaemia, while the non‐ischaemic limb is subjected to systemic inflammation without ischaemia. We hypothesized that the combination of ischaemia and inflammation in the surgical limb would induce infiltration of immune cells and systemic increases in levels of inflammatory cytokines, and that these inflammatory changes would induce immune infiltration in muscles of the non‐ischaemic limb, albeit to a lesser degree. We further hypothesized that these inflammatory changes in skeletal muscle would be accompanied by skeletal muscle atrophy and capillary rarefaction in both the ischaemic and non‐ischaemic limbs of the HLI mice compared with a Sham operation condition.

## 2 METHODS

### 2.1 Ethical approval

All animal research procedures described below are in compliance with the ‘Guide for the Care and Use of Laboratory Animals’, 8th Edition, and were approved (R‐JUL‐21‐50) by the University of Maryland Institutional Animal Care and Use Committee (IACUC).

### 2.2 Animals

**FIGURE 1:**
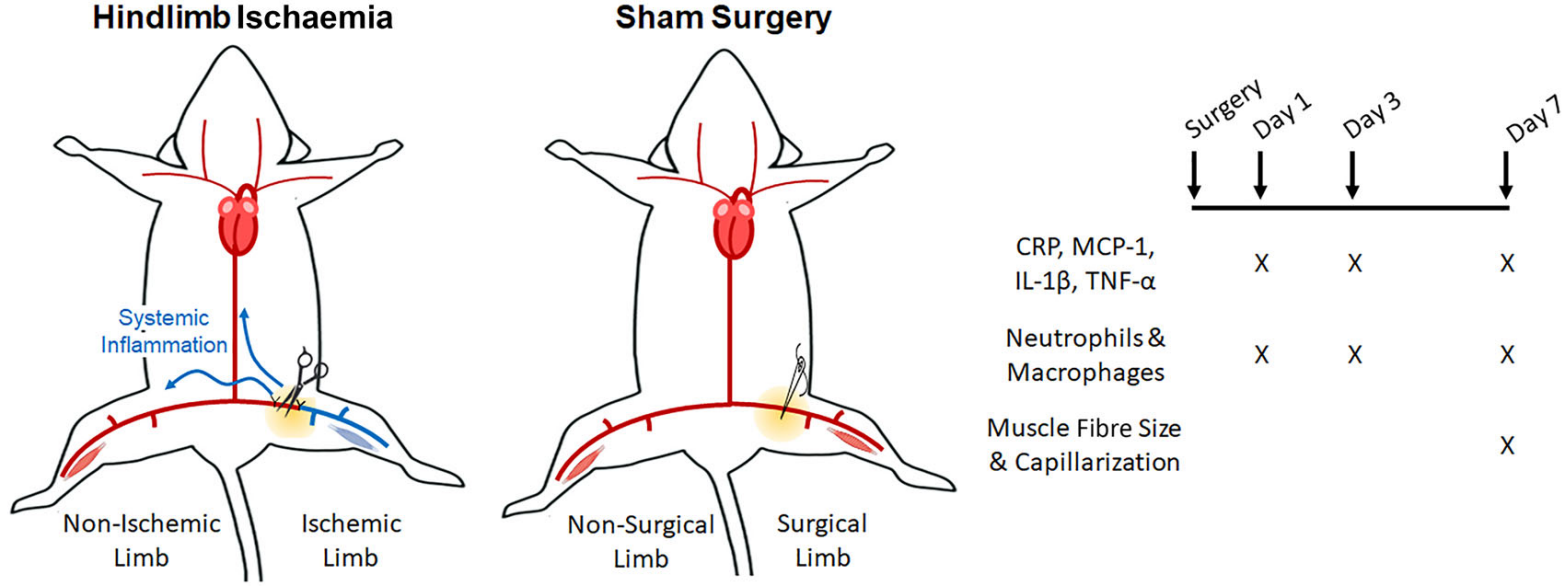
Experimental design. Eight‐week‐old male mice (C57BL/6NTac) underwent either hindlimb ischaemia (HLI) or Sham surgery and were killed at days 1, 3 or 7 after surgery. Both hindlimbs from each mouse were studied to determine the local and remote effects of HLI or Sham surgery on systemic inflammation and skeletal muscle phenotypes. Blood was collected at days 1, 3 and 7 for measurement of plasma concentrations of C‐reactive protein (CRP), monocyte chemoattractant protein‐1 (MCP‐1), interleukin 1β (IL‐1β), and tumour necrosis factor α (TNFα). Soleus muscles were harvested at each time point for measurement of skeletal muscle neutrophil and macrophage infiltration. Gastrocnemius muscles from day 7 were studied to determine any effects on skeletal muscle fibre size and capillarization.

Twenty six, 8‐week‐old male mice (C57BL/6NTac, Taconic Biosciences, Albany, NY, RRID:IMSR_TAC:B6) were used in this study. The experimental design is presented in Figure 1 and mouse characteristics are presented in Table 1.

**TABLE 1:** Mouse age at surgery and masses for HLI and Sham mice at days 1, 3 and 7.

	HLI			Sham		
	Day 1	Day 3	Day 7	Day 1	Day 3	Day 7
*n*	4	4	5	4	4	5
Age at surgery (weeks)	12.7 ± 1.7	10.8 ± 0.5	11.8 ± 0.9	11.7 ± 1.7	9.5 ± 0.1	10.3 ± 0.9
Mass (g)	25.0 ± 0.6	23.8 ± 1.8	23.2 ± 0.8	23.5 ± 1.7	24.5 ± 0.5	23.2 ± 1.0

*Note*: HLI and Sham procedures were performed 1, 3 and 7 days after surgery. Masses and mouse age at surgery are reported as means and standard errors. Abbreviation: HLI, hindlimb ischaemia.

### 2.3 HLI surgical procedures

Mice were anesthetized with isoflurane (2%–5% supplemented with 100% oxygen (400–500 mL/min)), weighed, placed on a warmed surgical table with isoflurane administration via nose cone (1.5%–2% supplemented with 100% oxygen (400–500 mL/min)), and buprenorphine (0.05–0.1 mg/kg) was administered via subcutaneous injection. Hair was removed from the surgical incision site and the site was cleaned and sterilized. A vertical skin incision was made ∼1 cm in length from the knee toward the medial thigh and mouse mid‐section. After dissection of subcutaneous fat and visualization of the femoral artery, skin was retracted, and the femoral artery was separated from the vein and nerve proximal to the caudal and popliteal arteries using a fine point cotton swab and fine forceps. For HLI, 7‐0 silk suture was passed around the femoral artery and two proximal knots were placed in tandem to occlude the vessel. Similarly, a suture was placed distal to the first occlusion, but proximal to the popliteal artery, leaving space for transection of the intervening segment with spring scissors. In Sham‐operated controls, the same procedures were followed, including the separation of the femoral artery from the nerve and vein, but the femoral artery was not occluded or transected. For both HLI and Sham surgeries, the incision was closed with a monofilament suture to close the dermal layer, and the site was again treated with betadine. Mice were given food and water ad libitum and monitored during recovery. A second dose of buprenorphine (0.05–0.1 mg/kg) was administered via subcutaneous injection within ∼12 h after surgery if mice showed signs of discomfort.

Mice were killed at 24 h (1 day), 72 h (3 days) and 7 days post‐surgery. Mice were anaesthetized with 2%–5% isoflurane supplemented with 100% oxygen (400–500 mL/min) as described above. After ceasing to respond to tail and toe pinches, a thoracotomy was performed, blood was collected via cardiac puncture, the heart was rapidly excised, and death was confirmed via exsanguination. Gastrocnemius muscles were excised for macrophage analyses and soleus muscles were excised for capillarization and fibre type analyses.

### 2.4 Skeletal muscle macrophage infiltration

To isolate macrophages, the entire gastrocnemius muscle was excised, and was dissected free of visible fat, tendons and fascia on ice. The gastrocnemius muscle was selected as it was large enough for digestion and enumeration of immune cells in our assays. Samples were minced and the entire gastrocnemius was digested in 50 mL conical tubes with the Skeletal Muscle Dissociation Kit (Miltenyi Biotec, Auburn, CA, cat. no. 130‐098‐305, RRID:SCR_020281) according to the manufacturer's guidelines. Briefly, samples were rotated at 37°C for 30 min and vortexed every 10 min to dislodge muscle pellets. During the next 30 min of digestion, samples were triturated with a wide bore pipette to aid in gentle mechanical digestion. Samples and buffers were placed on ice for the remainder of the procedure. Homogenates were passed through a 100 μm filter and washed with 10–15 mL of fresh serum‐free Dulbecco's modified Eagle's medium, then spun at 500 *g* for 20 min at 4°C to remove platelets and cell debris. Red blood cells were lysed, and samples were washed with cell staining buffer (CSB) containing 2% FBS, 10mM EDTA in Ca^2+^ and Mg^+^ free phosphate‐buffered saline (PBS). Homogenates were incubated with pre‐titrated volumes of anti‐rat FcR blocker (BD Biosciences, San Jose, CA, USA, cat. no. 550271, RRID:AB_393568) in CSB followed by staining with pre‐titrated amounts of 4′,6‐diamidino‐2‐phenylindole (DAPI; Thermo Fisher Scientific, Waltham, MA, USA), anti‐CD11b fluorescein isothiocyanate (Thermo Fisher Scientific cat. no.11‐0112‐82, RRID:AB_464935) and anti‐MHCII PerCP‐eFluor 710 (Thermo Fisher Scientific cat. no. 46‐0463‐82, RRID:AB_10736599). Samples were washed, passed through a 30‐μm filter, and run on a FACS Canto II (BD Biosciences) for cell counts to determine neutrophil and macrophage cell infiltration into the muscle. Appropriate single stain controls and fluorescence minus one (FMO) controls were run for compensation and gating. Doublets, debris and dead cells were gated out and a CD11b^+^ versus MHCII^+^ gate was used to determine different positive and negative subsets. Back gating of size and granularity was used on CD11b^+^ cells for neutrophil counts. The relative counts of CD11b^+^MHCII^+^ and CD11b^+^MHCII^−^ were compared.

### 2.5 Skeletal muscle capillarization and fibre size

After excision, the bellies of the soleus muscles from each limb were embedded in Tissue‐Tek Optimal Cutting Temperature (OCT) Compound (Sakura, Torrence, CA), frozen on liquid nitrogen, and stored at −80°C until analysis. Soleus muscles were selected for histology in order to obtain transverse sections of whole muscle for analyses. Samples were coded with unique identifiers so that investigators were blinded to limb and condition during subsequent analyses.

OCT‐embedded soleus muscle samples were placed in a cryostat at −20°C and mounted. Muscle orientation was determined and 10–15 μm transverse sections were cut and placed on 10 mm glass slides. Sections were fixed and permeabilized with ice cold acetone for 7 min and washed three times with PBS. After washes, tissue was blocked with 5% goat serum for 1 h at room temperature followed by three washes. *Griffonia simplicifolia* Lectin I Dylight 594 (Vector Laboratories, Newark, CA, USA, cat. no. DL‐1207, RRID:AB_2336415) (5 μg/mL), and rabbit anti‐Laminin (Abcam, Waltham, MA, USA, cat. no. AB_11575, RRID:AB_298179) (5 μg/mL) primary antibodies were added for 30 min at room temperature. After washing with PBS, secondary antibodies anti‐rabbit AF488 (Thermo Fisher Scientific, cat. no. A‐11034, RRID:AB_2576217) was added at 1 μg/mL for 30 min at room temperature followed by three washes with PBS (Prior et al., 2014). Sections were mounted with ProLong™ Gold Antifade Mountant with DAPI (Thermo Fisher Scientific) and immediately imaged with a Zeiss Axio Observer (Carl Zeiss Microscopy, White Plains, NY, RRID:SCR_021351) fluorescence microscope. Indices of capillarization and muscle morphology were determined, including (1) capillary contacts per fibre, (2) individual capillary to fibre ratio, (3) capillary density, (4) muscle fibre perimeter, and (5) muscle fibre cross‐sectional area, as previously published (Prior et al., 2014, 2009, 2015). These measurements were obtained using the Muscle2View (CellProfiler, Broad Institute of MIT, and Harvard) pipeline (Sanz et al., 2019). Data were validated by crosschecking post‐processing images with manual measurements.

### 2.6 Plasma cytokine concentrations

To determine the concentrations of inflammatory cytokines, plasma samples were thawed on ice and a customized multiplex assay was performed according to manufacturer guidelines (Mouse Procartaplex, Thermo Fisher Scientific) for tumour necrosis factor α (TNFα), interleukin 1β (IL‐1β), C‐reactive protein (CRP) and monocyte chemoattractant protein‐1 (MCP‐1). Samples were analysed on a custom Luminex MAGPIX (Luminex Corporation, Austin, TX, USA) multiplex plate reader and data are reported as median fluorescence intensity. Adequate blood for cytokine assays was obtained from four mice for day 1, 6 mice for day 3 and 10 mice for day 7 time points. One HLI day 7 sample was excluded due to errors while running the analytes on the Luminex, and one day 7 Sham IL‐1β reading was excluded due to a low bead count.

### 2.7 Statistical analyses

An independent Student's *t*‐test was used to compare variables between limbs within and between the HLI and Sham groups using GraphPad Prism v10.2.0 (GraphPad Software, Boston, MA, USA). A *P*‐value <0.05 was deemed statistically significant.

## 3 RESULTS

### 3.1 Animal characteristics

Age and body mass did not different between Sham and HLI mice; animal characteristics are shown in Table 1.

### 3.2 Systemic inflammatory cytokines

**FIGURE 2:**
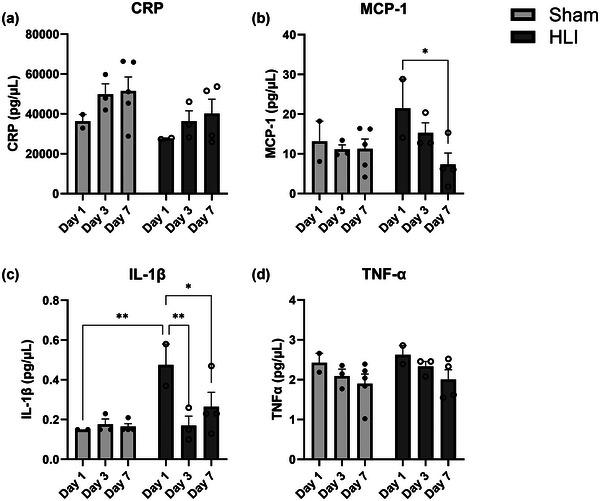
Systemic concentrations of IL‐1β and MCP‐1 respond to hindlimb ischaemia. Blood plasma was collected after day 1 (HLI *n* = 2; Sham *n* = 2), day 3 (HLI *n* = 3; Sham *n* = 3) and day 7 (HLI *n* = 4, Sham *n* = 5) and frozen. Samples were thawed and analysed with a multiplex bead assay to measure systemic concentrations of an acute phase reactant (a, CRP), a chemokine (b, MCP‐1), and proinflammatory cytokines (c, IL‐1β; d, TNFα) in Sham and HLI mice. Independent *t*‐tests were used to compare groups and time points per our a priori hypotheses. Data are presented as means and standard errors. *P < 0.05 for independent *t*‐tests. *P < 0.05, **P < 0.01, ***P < 0.001, ****P < 0.0001. CRP, C‐reactive protein; HLI, hindlimb ischaemia. IL‐1β, interleukin 1β; MCP‐1, monocyte chemoattractant protein‐1; TNFα, tumour necrosis factor α.

Plasma inflammatory cytokine concentrations were measured to determine the effect and time course of HLI or Sham surgery on circulating inflammatory markers associated with macrophage function and recruitment (Figure 2). IL‐1β concentrations peaked on day 1 following HLI, as mean IL‐1β concentration was 216% greater in HLI than Sham (*P* = 0.0046), then decreased at days 3 and 7 to levels similar to Sham mice. MCP‐1 concentrations appeared to be ∼100% higher in HLI than Sham at day 1; however, this did not reach statistical significance. Compared with day 1, MCP‐1 concentrations decreased in HLI mice by 29% and 73% at days 3 and 7 (*P* = 0.013), respectively, to levels similar to Sham. CRP and TNFα concentration were not different between HLI and Sham.

### 3.3 Muscle immune cell infiltration

**FIGURE 3:**
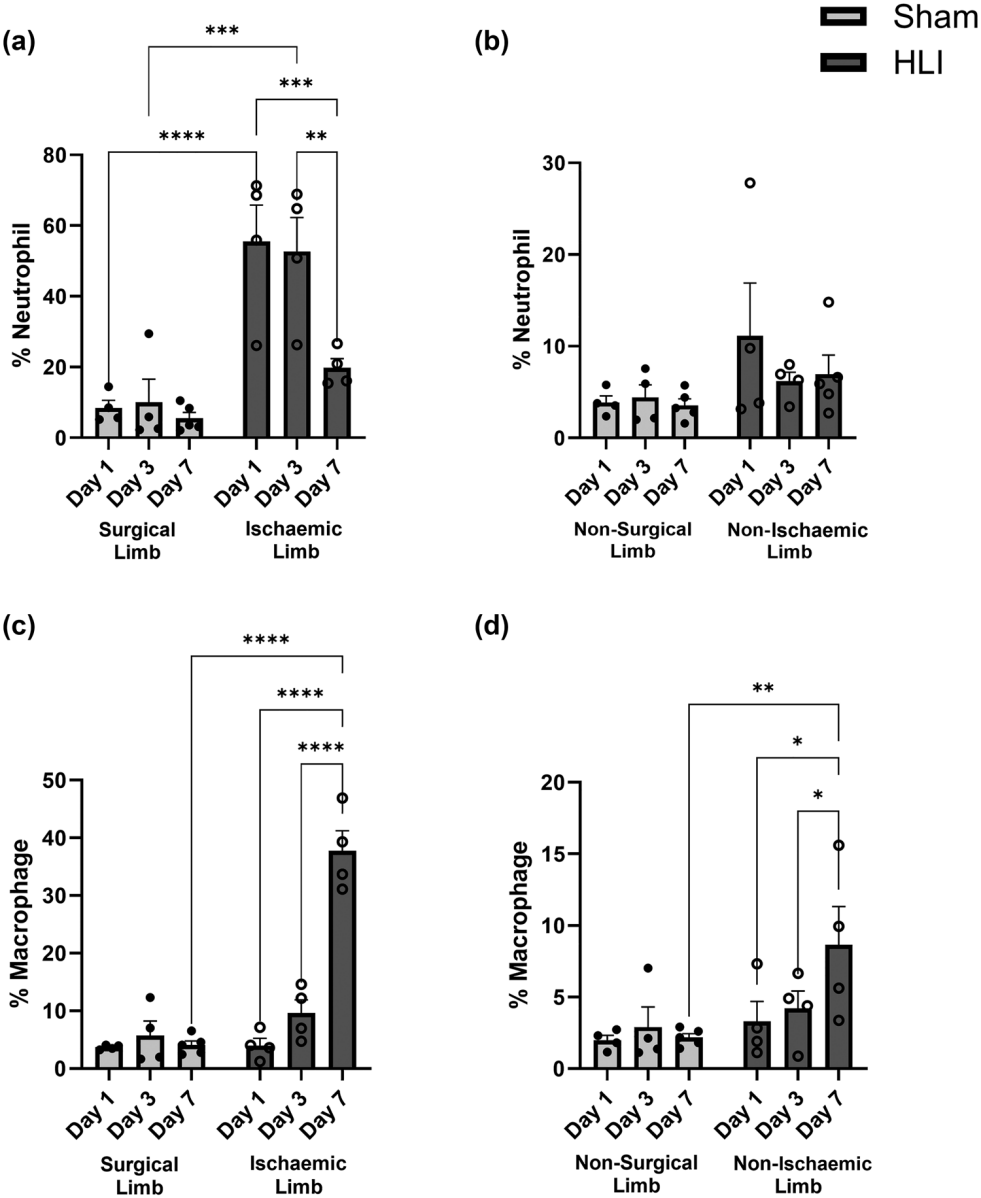
Skeletal muscle neutrophil and macrophage infiltrate in mice 7 days after unilateral hindlimb ischaemia or Sham. Gastrocnemius muscles were freshly digested from limbs of mice after an HLI or Sham procedure. Cells were stained against CD11b, DAPI and MHCII and the relative percentage of neutrophils (a, b) and macrophages (c, d) was determined by flow cytometry. A time course was performed by assessing muscle at day 1 (*n* = 4/group), day 3 (*n* = 4/group) and day 7 (*n* = 4–5/group) post‐surgery. Muscle infiltrate was compared with *t*‐tests and data are presented as means and standard errors. *P < 0.05, **P < 0.01, ***P < 0.001, ****P < 0.0001. CD11b, integrin α M; DAPI, 4′,6‐diamidino‐2‐phenylindole; HLI, hindlimb ischaemia; MHCII, major histocompatibility complex II.

Skeletal muscle macrophage and neutrophil infiltration were measured in both limbs of mice receiving HLI and Sham procedures to determine whether changes in skeletal muscle macrophages occurred in ischaemic and remote skeletal muscle in response to HLI. Compared with the Sham surgical limb, the gastrocnemius muscle from the ischaemic limbs of HLI mice had >5‐fold more neutrophils on day 1 (Figure 3a, *P *< 0.0001), and >4‐fold more neutrophils on day 3 (Figure 3a, *P* = 0.0001). This neutrophil infiltration decreased in ischaemic muscle from HLI mice at day 7 such that there was no significant difference from the surgical limb in Sham by day 7. Macrophage infiltration of muscle in the ischaemic limb from the HLI group did not differ from the surgical limb in Sham at days 1 or 3, but at day 7, there was a nearly 7‐fold greater number of macrophages in the muscle from the ischaemic limb of HLI compared with Sham (Figure 3c, *P *< 0.0001).

Similar trends in neutrophil and macrophage infiltration were present in muscle from the non‐ischaemic limb of HLI mice compared with the non‐surgical limb of Sham mice, but to a lesser degree. Neutrophil number appeared ∼2‐fold higher in HLI mice than Sham mice at day 1, with a subsequent decrease at days 3 and 7 in the HLI mice, but these differences did not reach statistical significance (Figure 3b). However, macrophage infiltration of muscle in the non‐ischaemic limb of HLI mice was significantly greater than the non‐surgical limb of Sham mice, with a 2.3‐fold greater number of macrophages in the muscle of HLI mice at day 7 compared with Sham mice (Figure 3d, *P *< 0.0001).

### 3.4 Muscle atrophy and capillarization

**FIGURE 4:**
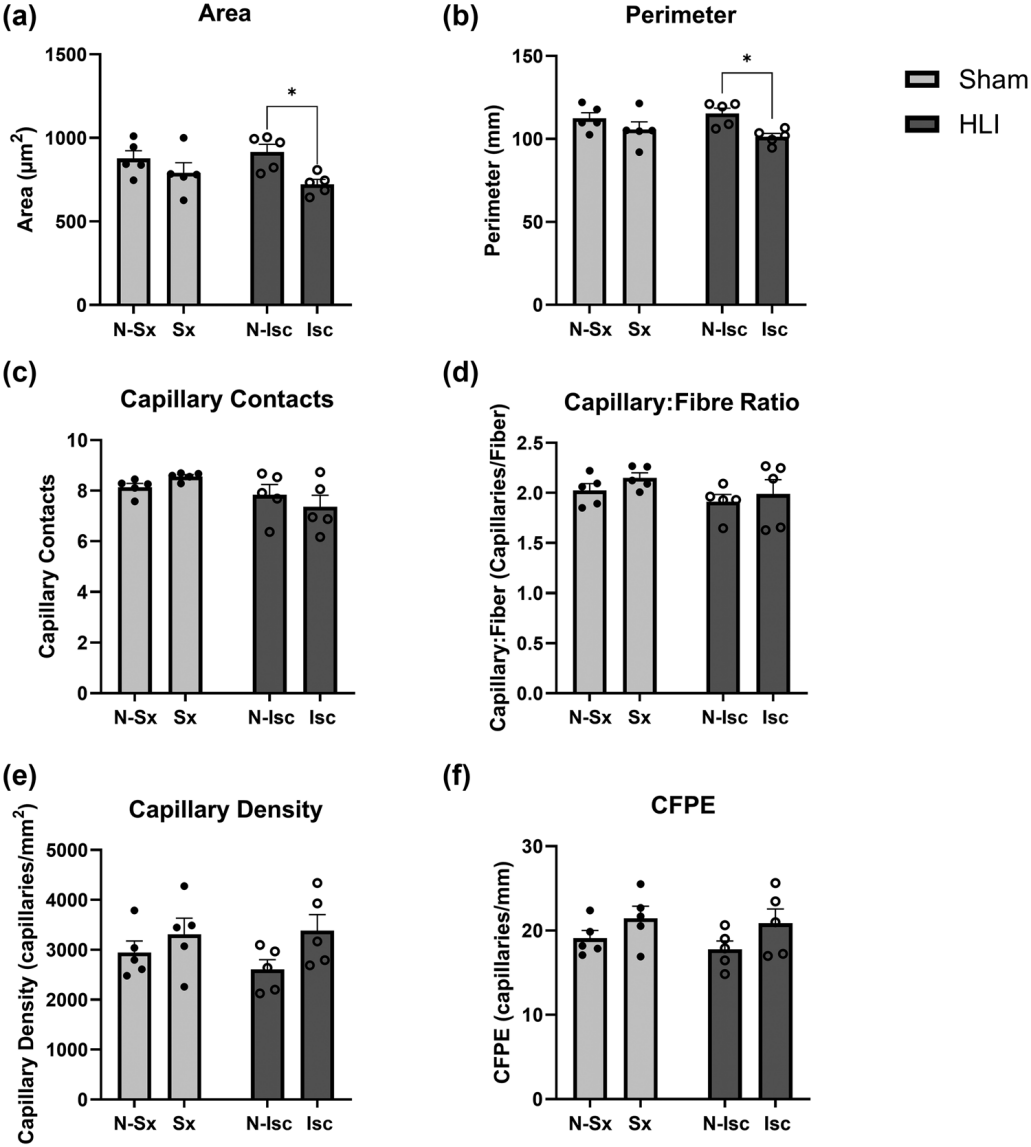
Inflammation did not significantly affect skeletal muscle fibre size and capillarization in the non‐surgical limbs of mice with hindlimb ischaemia after 7 days. Three separate 10 μm cross sections of the soleus from Sham (*n* = 5) or HLI (*n* = 5) mice were stained and imaged with a fluorescent microscope at 200x magnification. Images were analysed with Muscle2View and verified manually to measure skeletal muscle fibre area (a) and perimeter (b), capillary contacts per fibre (c), capillary‐to‐fibre ratio (d), capillary density (e), and capillary‐fibre perimeter exchange index (f). Data are represented as mean ± standard error of the mean. Independent *t*‐tests were used to compare limbs between HLI (*n* = 5) and Sham (*n* = 5) mice and paired *t*‐tests were used to compare limb differences within a condition. *P < 0.05, **P < 0.01, ***P < 0.001, ****P < 0.0001. CFPE, capillary‐fibre perimeter exchange index; HLI, hindlimb ischaemia; Isc, ischaemic limb; N‐Isc, non‐ischaemic limb; N‐Sx, non‐surgical limb; Sx, surgical limb.

We next determined whether the HLI surgery had significant effects on fibre atrophy or skeletal muscle capillarization in the surgical or remote muscle by histological staining (Figure 4). When comparing the limbs within HLI mice, the mean cross sectional muscle fibre area of the ischaemic limb was 21% smaller (Figure 4a, *P* = 0.01) and the mean fibre perimeter was 12% smaller (Figure 4b, *P* = 0.011) than the non‐ischaemic limb, indicating an effect of HLI on ischaemic muscle. Measures of capillarization and fibre size did not differ in the non‐ischaemic limb of HLI mice compared with either limb in the Sham mice, suggesting no short‐term effect of HLI on these variables in non‐ischaemic muscle.

## 4 DISCUSSION

The main finding of this study was that HLI induces acute, systemic inflammation with subsequent neutrophil infiltration of skeletal muscle that was followed by macrophage infiltration in both ischaemic and non‐ischaemic limbs of HLI mice. While macrophage infiltration of skeletal muscle occurred in both limbs of HLI mice, smaller muscle fibre size was only observed in the ischaemic limb; thus, the early effects on fibre size appear attributable mainly to ischaemia. These findings collectively demonstrate that HLI‐induced inflammation is sufficient to stimulate infiltration of immune cells in both ischaemic and remote skeletal muscle tissue. While this immune infiltration was not sufficient to induce skeletal muscle fibre atrophy in the non‐ischaemic limb of HLI mice over the 7‐day time course of this study, this may predispose skeletal muscle to subsequent inflammation‐induced injury and signal a priming of skeletal muscle atrophy over a longer time course or in response to future inflammatory challenges.

Previous work demonstrates that plasma concentrations of pro‐inflammatory cytokines such as IL‐6, IL‐1β, TNFα, CRP and MCP‐1 are elevated in chronic CVDs and are correlated with poor CVD outcomes (Hoogeveen et al., 2005; Ridker et al., 1997, 1998, 2001, 2002, 2017; Ridker & Lüscher, 2014; Ridker, Hennekens, et al., 2000; Ridker, Rifai, Pfeffer, et al., 2000; Ridker, Rifai, Stampfer, et al., 2000). Despite the well‐accepted hypothesis that systemic inflammation contributes to skeletal muscle atrophy and vascular rarefaction, there are a lack of basic studies demonstrating or investigating this phenomenon. In HLI models, researchers investigate the ischaemic limb relative to the contralateral, non‐ischaemic limb in the same animal to understand mechanisms related to ischaemia, hypoxia and angiogenesis; however, this presents a limitation because it is unknown if, and how, ischaemia in one limb may affect remote skeletal muscle. In the present study, we used a Sham condition as a comparison group to better understand the effects of HLI‐induced systemic inflammation on remote muscle. We expected that in HLI mice, the non‐ischaemic limb would only be subjected to the effects of systemic inflammation, while the ischaemic limb would experience the direct effects of both inflammation and ischaemia.

Of the cytokines measured, we observed significantly greater IL‐1β levels in HLI mice compared with Sham mice on day 1. MCP‐1 concentrations were 70% greater on day 1 than with Sham mice, but this did not reach significance. In both HLI and Sham controls, pro‐inflammatory cytokine concentrations generally were highest on day 1 and decreased thereafter, while acute phase reactant CRP concentrations tended to increase from day 1 to 7. This pattern of systemic inflammation was expected and is representative of an acute inflammatory response in both Sham and HLI, though to a greater extent in the HLI condition for MCP‐1 and IL‐1β. This rapid rise of cytokines such as MCP‐1 following surgery is responsible for rapid recruitment of monocytes to sites of tissue damage to prevent adverse remodelling. In fact, deletion of MCP‐1 in an HLI model delays monocyte appearance and increases necrotic tissue (Wang et al., 2018). The greater concentrations of IL‐1β in the HLI mice are notable, as this is a particularly potent inflammatory produced by neutrophils and macrophages, cell types that were demonstrably higher in the muscle of HLI versus Sham mice. Finally, the later increase in CRP in the absence of differences in the HLI and Sham condition is unsurprising given that it is a downstream marker of inflammation following stimulation of the liver with IL‐6. Moreover, CRP is largely regarded as an important marker of chronic inflammation, while our model was investigated to study the acute temporal nature of inflammation and its effects on remote immune cells and skeletal muscle. Though we expected to find differences in all measured inflammatory cytokines between the HLI and Sham conditions, the lack of some differences between groups for CRP and TNFα could be attributable to inflammatory effects of the Sham surgery. Inflammatory cytokines are sensitive markers of inflammation, and even successful surgeries are accompanied by elevations in plasma inflammatory cytokines for multiple days (Roth‐Isigkeit et al., 1999). As our Sham surgery included an incision and separation of the artery, nerve and vein, this perturbation likely induced an inflammatory response, and levels of some cytokines could have been elevated above basal.

With regard to the immune infiltration of the ischaemic limb in HLI mice, we saw a large influx of myeloid cells on day 1 as >60% of all immune cells were CD11b^+^ and had size and scattering properties of neutrophils compared with ∼10% in the Sham condition. The neutrophil count decreased and was similar to Sham mice by day 7, but the macrophage counts gradually increased from day 1 to 7 in the HLI ischaemic limb, which is consistent with monocytic infiltration and subsequent macrophage differentiation. This large influx of immune cells may be attributable to local production of cytokines involved in chemotaxis by ischaemic muscle. Skeletal muscle is known to produce the cytokines measured in this study (IL‐1β, MCP‐1 and TNFα) (Catoire et al., 2014; Jensen et al., 2020; Lang et al., 2003) in addition to other chemoattractant cytokines such as toll‐like receptor 4 (TLR4) (Drummond et al., 2013). It is likely that the skeletal muscle expression of these chemoattractants was increased in ischaemic muscle and could explain the higher neutrophil and macrophage infiltration compared with non‐ischaemic muscle.

While we expected robust effects in the ischaemic limb of HLI mice, we also hypothesized that the systemic inflammatory response would contribute to an immune inflammatory response in the non‐ischaemic limb. We found the timing of this response in the ischaemic and non‐ischaemic limbs to be similar, with neutrophils peaking on day 1 then decreasing, while macrophages were similar on day 1 before gradually increasing in the HLI condition. In the non‐ischaemic limb of HLI mice, there were ∼2.3‐fold more macrophages by day 7 than in the non‐surgical limb in Sham condition. This is a substantial increase and represents an important feature of the systemic inflammatory response. In fact, the relative percentage of macrophages in the non‐ischaemic limb of HLI mice was similar to the surgical limb in the Sham condition; therefore, the systemic changes in HLI mice resulted in similar concentrations of immune cells in non‐ischaemic muscle as making a physical incision and perturbing the underlying tissue in the surgical limb of Sham mice. This is important because the latter is accompanied by wound repair and tissue remodelling, while the former is occurring absent any structural changes. Moreover, the increase in macrophages in skeletal muscle of the non‐ischaemic limb of HLI mice is interesting because macrophages are tissue resident cells that *typically* originate from monocyte differentiation (Gomez Perdiguero et al., 2015). We cannot be certain, but it is possible that these macrophages were sloughed off the ischaemic limb and circulated, or recruited via MCP‐1, as there was numerically more MCP‐1, a potent monocyte chemokine, on day 1 after HLI compared with Sham. Regardless of their origin, these cells could have functional ramifications that place the muscle in a ‘primed’ state (Sorrentino et al., 2018).

We also hypothesized that skeletal muscle fibre size and capillarization would be reduced in the non‐ischaemic limb of HLI mice compared with the non‐surgical limb of Sham mice due to the observed immune inflammatory response; however, there were no differences in skeletal muscle fibre size or capillarization of the non‐ischaemic limb between HLI and either limb in Sham mice. We posited that capillarization and fibre size could be influenced by heightened systemic and local concentrations of white blood cells that produce pro‐inflammatory cytokines. These cytokines are known to impede myoblast proliferation and differentiation, and chronic exposure of endothelial cells to pro‐inflammatory cytokines like TNFα has been observed to hinder endothelial angiogenesis (Chen et al., 2007; Sainson et al., 2008). Additionally, MCP‐1 plays a role in recruiting muscle macrophages, which when uncontrolled has been linked to delayed angiogenesis and hindered muscle regeneration (Shireman et al., 2007). Moreover, activation of IL‐1β in muscle tissues has been associated with increased collagen accumulation, contributing to muscle fibrosis (Segawa et al., 2008). However, in studies showing elevated plasma concentrations of inflammatory cytokines and disease, the elevations are chronic, often accumulating over many years (Ridker et al., 2000a). In our study, changes in muscle morphology were only measured after 7 days, so it may be possible that a longer time course would elicit differences in muscle fibre size and capillarization. The durability of capillarization is supported by studies of exercise cessation, wherein exercise training‐induced increases in muscle capillarization are maintained after 2 weeks of detraining (Prior et al., 2015), before decreasing after >8 weeks of detraining (Klausen et al., 1981). Another possibility is that the magnitude of differences in cytokine concentrations between HLI and Sham mice may not have been sufficient to elicit tissue changes, as 0.5 or 5 ng of TNFα, but not 0.05 ng of TNFα was required to impair myoblast proliferation and differentiation in cell culture (Miller et al., 1988).

Despite the strengths and unique aspects of our study assessing both the ischaemic and non‐ischaemic limbs of HLI mice, the study is not without limitations. First, experiments were only conducted in male mice, so future experiments should be conducted in female mice to determine if sex‐specific differences exist. Second, while this initial study was performed in young mice, future experiments need to be conducted in older animals to determine if responses are more robust given the heightened incidence of CVDs and systemic inflammation in ageing. Additionally, our experiments investigated muscle capillarization and fibre size, but there could be functional changes in endothelial cells that may also present in the non‐ischaemic limb of our HLI mice. Furthermore, the gastrocnemius and soleus muscles have different proportions of fibre types that could possibly influence the production of chemoattractant cytokines. While we saw substantial immune infiltration in gastrocnemius muscles, we cannot say for certain whether the same immune infiltration occurred in soleus muscle, in which capillarization and fibre type were measured. Finally, we measured systemic inflammatory cytokines, but assessments of the circulating immune cell fraction would aid in our understanding of the systemic response.

In the initial 7‐day period following HLI, neither skeletal muscle fibre size nor capillarization decreased in the non‐ischaemic limb, but there was an increase in circulating inflammatory cytokine levels and an increased presence of skeletal muscle macrophages in both the ischaemic and non‐ischaemic skeletal muscle in HLI compared with Sham. This study highlights the potential utility of HLI as a model for studying the effects of acute systemic inflammation on neutrophil and macrophage accumulation in the non‐ischaemic tissues, as well as ischaemic tissues. We speculate that these immune cells have functional implications that unfold over a longer period of time than the 7‐day time course of the present study, and that subsequent or prolonged bouts of systemic inflammation could lead to morphological changes and functional decline. These findings demonstrate the feasibility of this model for future, in‐depth investigations into systemic inflammation, the origin of macrophages infiltrating skeletal muscle, and their phenotype and effects on skeletal muscle.

## AUTHOR CONTRIBUTIONS

Conception or design of the work: William S. Evans, Sarah Kuzmiak‐Glancy, Steven J. Prior. Acquisition, analysis, or interpretation of data for the work: William S. Evans, Gabriel Pena, Beata Gelman, Sarah Kuzmiak‐Glancy and Steven J. Prior. Drafting of the work or revising it critically for important intellectual content: William S. Evans, Gabriel Pena, Beata Gelman, Sarah Kuzmiak‐Glancy and Steven J. Prior. All authors approved the final version of the manuscript and agree to be accountable for all aspects of the work in ensuring that questions related to the accuracy or integrity of any part of the work are appropriately investigated and resolved. All persons designated as authors qualify for authorship and all those who qualify for authorship are listed.

## CONFLICT OF INTEREST

The authors have no conflicts of interest to disclose.

## Data Availability

Supporting data are available upon request.
